# Reduced Image Quality Impairs Monocular Blur-Driven Accommodation in Keratoconus

**DOI:** 10.1167/iovs.67.6.52

**Published:** 2026-06-26

**Authors:** Tithi Bhakta, Ketakee Jain, Priyanka Sudanabonia, Santiago Sager, Adrian Gambin, Pablo Artal, Shrikant R. Bharadwaj

**Affiliations:** 1 Brien Holden Eye Research Centre, L. V. Prasad Eye Institute, Telangana, India; 2 Brien Holden Institute of Optometry and Vision Sciences, L. V. Prasad Eye Institute, Telangana, India; 3 Cataract and Refractive Services, L. V. Prasad Eye Institute, Hyderabad, Telangana, India; 4 Laboratorio de Optica, Universidad de Murcia, Murcia (Región de Murcia), Spain; 5 Voptica, Universidad de Murcia, Murcia (Región de Murcia), Spain

**Keywords:** amplitude spectrum, contrast loss, higher-order aberrations (HOAs), image quality, phase shift

## Abstract

**Purpose:**

The purpose of this study was to test the hypotheses that (1) monocular blur-driven accommodation is attenuated in keratoconus due to weaker defocus-blur signals arising from degraded image quality (IQ) compared with non-keratoconic controls, and (2) manipulating the strength of defocus-blur signal, either by correcting higher-order aberrations (HOAs) in keratoconus or by inducing keratoconic HOAs in controls, will correspondingly improve or degrade accommodative performance.

**Methods:**

Monochromatic IQ degradation was computed from HOAs of 15 mild-to-moderate keratoconic participants (13–29 years) and 15 controls for a 4.5-mm pupil diameter. Participants monocularly accommodated to 0.5 to 4.0 diopter (D) hyperopic step changes whereas their lower-order aberrations (LOAs) or both LOAs and HOAs were corrected using an adaptive optics visual simulator. The protocol was repeated in 10 controls with induced HOAs from one moderate keratoconic eye.

**Results:**

IQ loss with induced blur was significantly lower in patients with keratoconus than in healthy controls (*P* < 0.001). Accommodative responses were reduced in keratoconus (0.6 ± 0.9 D across the 4-D range) compared with controls (2.6 ± 1.1 D, *P* < 0.01) and were correlated with the corresponding IQ loss (*r* = 0.75, *P* < 0.001). Although IQ improved with combined LOA + HOA correction in keratoconus, no significant change in accommodation was observed (*P* = 0.58). Inducing keratoconic HOAs, however, significantly attenuated accommodation in controls (0.27 ± 0.41 D, *P* = 0.001).

**Conclusions:**

Reduced monocular accommodation is consistent with weakened defocus-blur signals available to drive these responses in keratoconic eyes. The absence of improvement in accommodation despite optical quality enhancement may indicate a limited role of pure defocus as an accommodative cue and/or reduced neural sensitivity to blur following chronically degraded retinal images.

Ocular accommodation is thought to be driven primarily by optical blur arising from the object of interest not being optically conjugate with the retina.^1^ This optical blur may, however, be a weak and unreliable cue for accommodation in highly aberrated eyes. This is indicated by the poorer image quality (IQ) of such eyes at best focus and a smaller change in IQ per unit change in dioptric blur, all relative to eyes with physiologically healthy optics.^2^^–^^5^ Indeed, the gain of blur-driven accommodation has been shown to decrease with induced positive spherical aberration or coma in eyes with otherwise healthy optics.^6^ It is possible that such an effect occurs naturally in keratoconus due to the degraded IQ arising from increased irregular astigmatism and higher-order wavefront aberrations (HOAs; especially, spherical aberration, coma, and trefoil; Figs. 1A, 1B).^7^^–^^9^ This study tested two hypotheses in this context. First, monocular blur-driven accommodation will be attenuated in keratoconus due to the weaker blur signals arising from the optical quality degraded by increased HOAs, vis-à-vis, non-keratoconic controls (Fig. 1C). Second, manipulating the strength of the blur signal either by correcting HOAs in keratoconus or by inducing keratoconic HOAs in controls will correspondingly improve or degrade accommodative performance, respectively. The improvement in accommodative performance following HOA correction will be larger in keratoconus, compared with controls (see Fig. 1C). On the other hand, the accommodative performance of controls is expected to mimic the behavior of cases with induced keratoconic HOAs (see Fig. 1C). Alternatively, the improvement of accommodative performance in keratoconus following HOA correction may be limited by the neural insensitivity to blur arising from chronic exposure to degraded optics. Such a possibility has been demonstrated for visual acuity changes before and after correction of HOAs in these eyes, in a study by Sabesan and Yoon (2009).^10^

**Figure 1.**
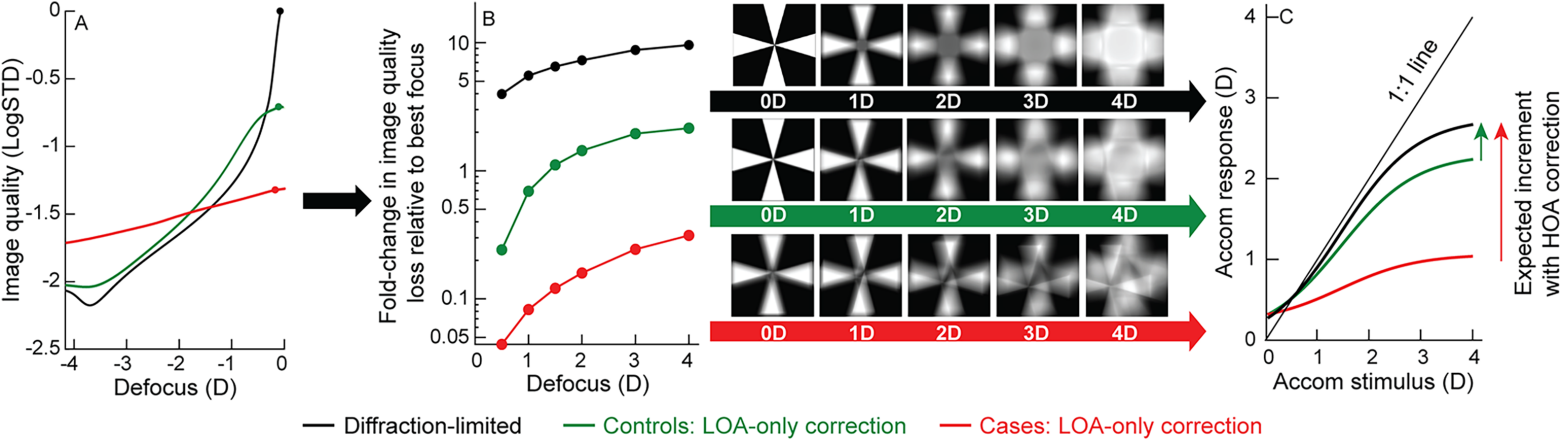
Image quality (IQ) analysis performed to determine the impact of higher-order wavefront aberrations (HOAs) on monocular, blur-driven accommodation using the profiles of 30 controls with physiologically normal optics and 30 cases with mild to severe keratoconus obtained from Nilagiri et al. (2020).^9^ All simulations were performed for 4.5-mm pupil diameter and 555 nm of light using standard Fourier optics techniques.^18^ (**A**) Averaged through-focus curves derived for the logSTD – logarithm of the standard deviation of the point spread function intensity, normalized to the diffraction-limited value – IQ metric for controls (*green curve*) and cases (*red curve*) with only their lower-order aberrations corrected (LOA-only corrected condition) or with both LOAs and HOAs corrected (LOA + HOA corrected condition; *black curve*). The keratoconic through-focus curve shows lower values of best IQ than in controls (−0.61 ± 0.09 and −0.99 ± 0.18 LogSTD in controls and cases, respectively) and the persistence of the sub-optical IQ for an extended dioptric range. (**B**) The loss in IQ with 0.5 to 4 D of induced blur for the different optical conditions in panel **A** (see Equation 1 for IQ loss calculation). This panel also shows a visual representation of how the accommodative target changes in appearance with different blurs across these optical conditions tested here. (**C**) A schematic of the accommodative stimulus-response function in controls and cases under the different optical conditions tested here. This function may be attenuated more in keratoconus by weakened blur signal, relative to controls. The improvement in this function with the correction of LOAs and HOAs may also be larger in cases than in controls.

Few studies have explored accommodative behavior in keratoconus but none from the perspective presented above. Ohmi et al.^11^ objectively demonstrated the maximum accommodative amplitude to be lower in keratoconus than in age-similar controls. In contrast, Dandapani et al.^12^ reported no difference in the subjective near point of accommodation in keratoconics and controls. The one study that measured blur-driven accommodation in keratoconus using a Shack-Hartmann aberrometer only compared results between two types of rigid contact lens correction, sans any data from non-keratoconic controls or data in keratoconics with spectacle wear.^13^ The former issue precludes any direct inference of accommodative deficiency in keratoconus, even while their data showed larger than expected accommodative lags in this cohort. Contact lens wear tends to minimize wavefront aberrations in keratoconus by replacing the distorted cornea with a uniform refracting surface.^14^^,^^15^ Therefore, their study does not allow an investigation on how the native optical degradation impacts accommodative behavior in keratoconus. Two other studies, available only in abstract form, report optical power fluctuations in keratoconus, but these are tangential to the present work.^16^^,^^17^ Overall, the literature does not offer any direct evidence in favor of or against the hypotheses set forth in the present study.

## Methods

### Participants

This prospective, case-control study, conducted at the L. V. Prasad Eye Institute (LVPEI), Hyderabad, India, involved 15 participants with mild to moderate keratoconus (mean ± 1 SD age = 20.1 ± 5 years, 9 male subjects) and 15 non-keratoconic controls (25.2 ± 2 years, 4 male subjects; Table 1). The study adhered to the tenets of the Declaration of Helsinki, and it was duly approved by the Institutional Review Board of LVPEI. All adults signed a written informed consent form, and a parent or legal guardian signed the written informed consent form for minors prior to study commencement. Participants were assigned a code with increasing numerosity order based on when they were approached for participation. The numerosity codes were not re-used if the subject declined to participate.

**Table 1.** Demographic and Clinical Details of all Keratoconic Study Participants

Participant	Age, Y	Sex	SER, D	D-index, unitless	HORMS, µm
KC1	28.0	F	−1.50	6.2	0.6
KC4	29.0	M	−1.00	7.6	0.4
KC5	18.0	M	−0.63	4.9	0.5
KC6	19.0	M	−3.00	8.1	1.0
KC7	18.0	M	−2.63	8.4	1.2
KC8	14.0	F	−1.25	4.7	0.4
KC9	13.0	F	0.00	3.5	0.2
KC10	27.0	F	−1.00	7.0	1.0
KC11	21.0	M	−1.00	7.4	0.8
KC12	24.0	M	−6.50	7.8	1.1
KC13	22.0	M	−1.00	7.5	0.9
KC14	16.0	M	−2.25	3.0	0.8
KC15	20.0	F	−3.63	4.8	0.8
KC16	18.0	F	−0.50	5.0	1.6
KC17	15.0	M	0.00	5.0	0.6
Cases, Avg ± 1 SD	20.1 ± 5.0	9 M : 6 F	−1.7 ± 1.7	6.1 ± 1.8	0.8 ± 0.4
Controls, Avg ± 1 SD [Min − Max]	25.2 ± 2.3 [22 to 29]	4 M : 11 F [−]	−0.5 ± 1.00 [−3.50 to 0.13]	1.4 ± 0.9 [−0.19 to 2.87]	0.2 ± 0.1 [0.13 to 0.41]
*P* value	0.002	–	0.021	<0.001	<0.001

D-index, tomographic measure of keratoconus severity; F, female; HORMS, root mean squared deviation of the higher-order wavefront aberrations (µm) over 4.5 mm pupil diameter; M, male; SER, spherical equivalent of refraction (D).

The mean (±1 SD) values of the relevant parameters from cases and comparison values of controls are also shown in this table.

The *P* values represent the output of two-tailed, heteroscedastic *t*-tests comparing the results of controls and cases.

All cases were clinically diagnosed to have keratoconus in one or both eyes by an expert ophthalmologist. Cases with advanced keratoconus, corneal scars, and with any other ocular or systemic co-morbidities were excluded. All cases wore habitual sphero-cylindrical spectacles, with best-corrected monocular visual acuity of 20/20 or better in both eyes. Habitual rigid contact lens wearers were excluded as this may influence the participant's habitual visual experience, thereby confounding the measurements of accommodation. Keratoconus severity was determined using the D-index from the Belin–Ambrósio Enhanced Ectasia Display map in the WaveLight Oculyzer II topography system (Alcon, Fort Worth, TX, USA; see Table 1).^19^ Controls were free of any ophthalmic or binocular vision dysfunction and had best-corrected monocular visual acuity of 20/20 or better in both eyes. Four controls were myopic (mean ± 1 SD spherical equivalent refractive error = −1.69 ± 1.34 diopter [D]) and 11 were emmetropic (−0.02 ± 0.09 D). All but two controls were naïve to the experiment.

### Stimulation and Measurement of Blur-Driven Accommodation

**Figure 2.**
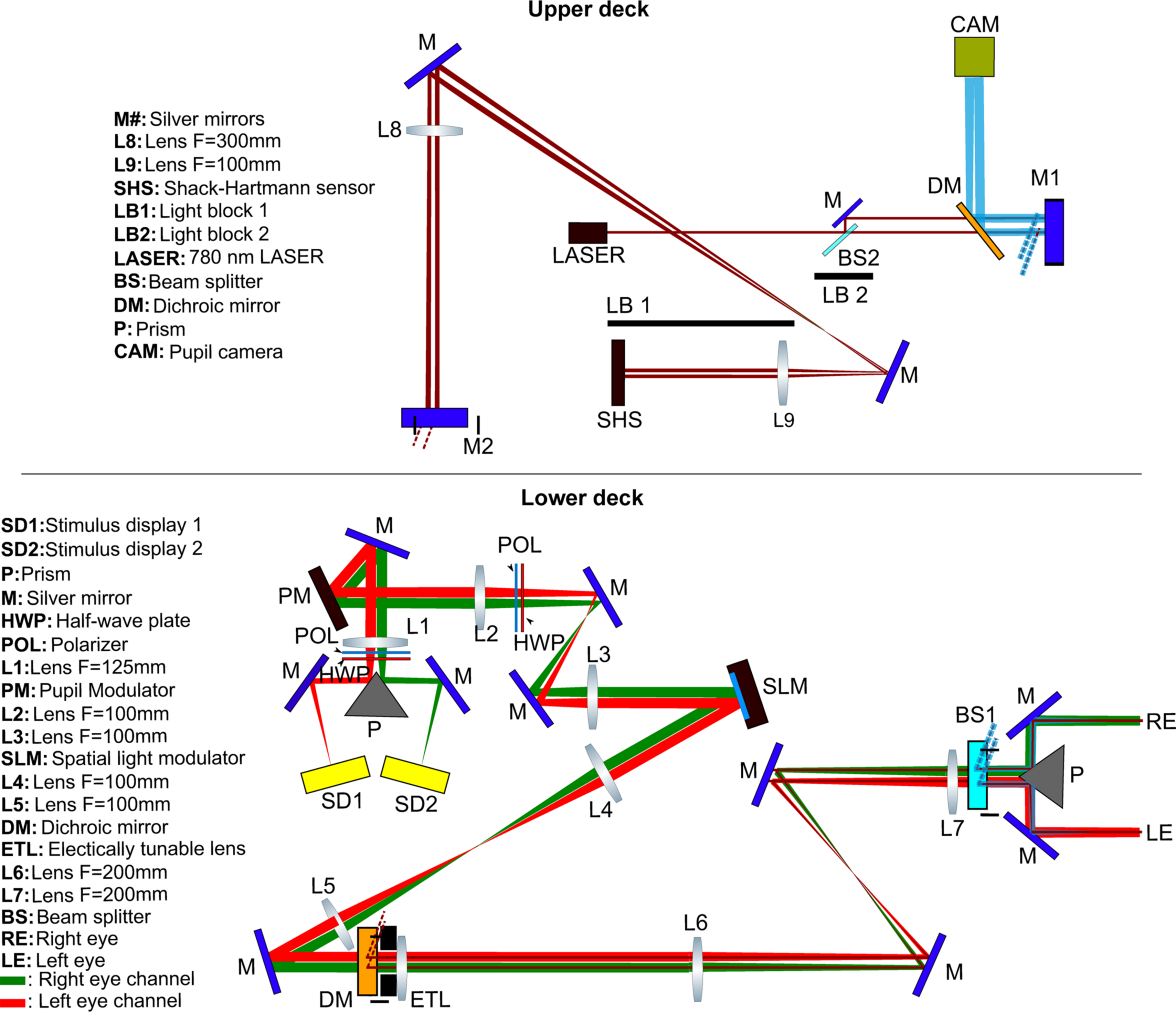
Schematic diagram of the two-tier binocular adaptive optics visual simulator used for stimulating and measuring accommodation in this study. The key components of the device are highlighted in the figure.

The study was conducted using a binocular adaptive optics visual simulator, originally described by Sager et al.^20^ and subsequently custom optimized for the measurement and correction of wavefront aberrations in keratoconus (Fig. 2). The measurement and correction channel in this device constituted provisions for subject positioning, pupil position and pupil size tracking, and for the measurement/correction of wavefront aberrations using a standard Shack-Hartmann sensor at the pupil plane. Wavefront corrections are applied through a spatial light modulator in the same plane. The second channel in the device is for psychophysical measurements, which includes two LCoS micro-displays positioned at optical infinity (i.e. 0 D of optical defocus) and an intensity modulator to create the pupils corresponding to each screen for eye-independent stimulus presentation. This channel is integrated with Matlab's Psychtoolbox software interface for stimulus presentation and psychophysics.^21^ The LCoS micro-displays are viewed by the subject through the focus-tunable lens and the spatial light modulator, ensuring that any changes in the optical pathway (i.e. correction or induction of wavefront aberrations) had a direct impact on the quality on the visual stimuli displayed. The Shack-Hartmann aberrometer is placed orthogonal to the correction channel such that the measurement of the eye's wavefront aberrations can be performed independent of its correction. The sensor measured the eye's wavefront aberrations at 780 nm from third to the 20th Zernike polynomial terms (i.e. up to the fifth order of Zernike polynomials), following the indexing convention of Optica.^22^ The error in defocus estimates arising in the wavefront sensor from longitudinal chromatic aberrations were corrected using a constant offset of 0.8 D in the device software.^20^ A detailed explanation on the effect of chromatic aberration for the spatial light modulator acting as phase modulators can be found in Martinez et al.^23^

These optical elements were arranged in two decks, as shown in Figure 2. These decks were connected by the measurement laser moving from the upper deck to the lower deck through mirror M1 in the upper deck to the beamsplitter BS1 in the lower deck. The light collected from the retina goes to the Shack-Hartmann sensor from the dichroic mirror DM in the lower deck to the mirror M2 in the upper deck. The combined range of defocus measurement and correction by the Shack-Hartmann sensor and the spatial light modulator is up to 16 D, large enough to correct the refractive errors and higher-order aberrations encountered in keratoconic cases (see Table 1).^24^ This device, however, does not perform closed-loop correction of wavefront aberrations and assumes that the wavefront aberrations and its correction remained stable throughout the experiment. This was periodically ensured throughout the current experiment. Although these functionalities may be applied to each eye individually, the present study was performed only on the left eye while the right eye remained occluded.

The participant was aligned with the device until a clear image of the pupil was obtained by the examiner. The pupil diameter was then fixed at 4.5 mm for the measurement and correction of wavefront aberrations. Participants fixated on a high-contrast black and white Maltese cross target rendered on the stimulus display while a spot-pattern was generated by the Shack–Hartmann sensor. Care was taken to maximize the number of spots within the processing pupil without overlap into adjacent spots. This spot pattern was used to optimize the participant's objective sphero-cylindrical refraction using the spatial light modulator following the standard convention of maximum plus power for maximum visual acuity. Blur-driven accommodation corresponding to 0.5, 1, 1.5, 2, 3, and 4 D was then stimulated over the 4.5 mm pupil diameter by varying the Zernike defocus term input into the correction channel (in microns, following diopter to micron conversion using standard formulae^25^). The stimulation followed a pseudo-random sequence for each participant, with each accommodative demand presented thrice for 6-seconds each and with an inter-stimulus interval randomly varying between 6 and 10 seconds to avoid anticipation. Shack–Hartmann spot patterns were obtained before stimulation of accommodation and at the end of each 6-second stimulation epoch to derive the pre- and post-stimulated Zernike polynomial coefficients, respectively. Although the analysis of the spot patterns for controls eyes could be easily automated, keratoconic eyes can result in a more asymmetric distribution of the spots in the Shack-Hartmann sensor, resulting in a less robust automatic detection for processing the pupil’s center. Hence, the position of the processing pupil was selected manually across two masked examiners and their findings were averaged (intraclass correlation coefficient = 0.89 [0.87–0.90], *P* < 0.001). Accommodation-related pupil miosis was avoided by dilating the pupils with 2.5% Phenylephrine HCl eye drops at the start of the experiment. This drug concentration has minimal to no influence on the eye's accommodative response.^26^

Accommodative responses were measured to defocus stimuli following correction of only the eye's defocus and astigmatism terms (lower-order aberrations [LOA]-only corrected) and following correction of both LOA and HOAs (LOA + HOA corrected). The latter was achieved by applying the inverse of the entire higher-order wavefront aberration terms to the spatial light modulator. The order of testing was counterbalanced across participants. An additional experiment was conducted on 10 controls by measuring accommodation following induction of HOAs of a keratoconic participant (see KC12 in Table 1). The keratoconic HOAs were added after correcting all aberrations of the controls. In all experiments, the participants were explicitly instructed to achieve and maintain clear vision of the Maltese cross target once it appeared blurred. Data collection lasted over 4 hours for each participant, with sufficient breaks provided to avoid fatigue and boredom.

### Assessment of Image Quality

Through-focus curves were computed for the LOA-only corrected and the LOA + HOA corrected conditions over 0 to 5 D of hyperopic defocus for 555 nm light and 4.5 mm pupil diameter using the logSTD metric to determine the extent of IQ loss before initiation of accommodation (see Fig. 1A).^9^ The HOA terms remained in the relaxed state for this analysis. The STD metric describes IQ as the SD of the point spread function intensity, normalized to the diffraction-limited value.^27^^,^^28^ This metric assumes a value of 1.0 [log_10_(1) = 0] when the IQ equals the diffraction-limited value. Negative values of this metric indicate worsening of IQ in the logarithmic space (see Fig. 1A). IQ loss for each value of induced defocus, relative to peak IQ, was calculated from the through-focus curve using Equation 1. The peak IQ, dioptric location of this peak (i.e. best focus) and the dioptric range over which the IQ remains within 80% of peak IQ (i.e. depth of focus) were computed from these through-focus curves.^9^ Through-focus curves for other IQ metrics that are as well-correlated with visual acuity in keratoconus yielded similar trends (e.g. NS and VSX^28^; data not shown).

(1) $$\begin{array}{c} \mathrm{IQ}\;loss=\frac{\mathrm{IQ}\;value_{defocus}-Peak\;IQ}{Peak\;IQ} \end{array}$$

Where, IQ loss = Loss in image quality for a given magnitude of defocus, IQ value_defocus_ = Value of the logSTD metric for a given defocus input in the LOA − only corrected through − focus curve, Peak IQ = Highest value of the logSTD metric in the LOA − only corrected through − focus curve.

### Data Analyses

Data analyses were performed using MS Excel (Microsoft Corporation, Redmond, WA, USA), MATLAB R2024a (MathWorks, Hyderabad, India), and IBM SPSS Statistics (version 21; Armonk, NY, USA). A difference in the pre- and post-stimulated Zernike defocus term obtained from the Shack-Hartmann sensor was considered as the surrogate measure of the eye's accommodative response, following microns to diopters conversion for 4.5 mm pupil diameter using standard conversion formulae.^25^ In addition to defocus, spherical aberration also changes with accommodation, the magnitude of which is correlated with the level of accommodative demand/response.^29^^–^^32^ A similar trend was observed in this study, more so for controls than cases (see Appendix I for details). However, because the defocus term remained the dominant signal, only its changes were deemed as a measure of the accommodative response in this study.^32^ Accommodative stimulus-response functions were fit using a five-parameter sigmoid function using standard nonlinear regression analysis (Equation 2). Because these *r*^2^ values of these fits were less than optimal for 6 of 15 cases and for 1 control (*r*^2^ ≤ 0.39), no further analysis was undertaken using these fits. These fits primarily served to make qualitative judgments about the accommodative response obtained across blur stimuli in this study.

(2) $$\begin{array}{ccc} & & \;Accom_{resp}=\\ & & \;\;\frac{Max\;accom_{resp}}{\left[1+\exp\left\{-slope\;*\;\left(Accom_{stim}-Accom\;stim_{mid\;point}\right)\right\}\right]}\; \end{array}$$

Where, Accom_resp_ = Accommodative response for a given accommodative stimulus (D), Max accom_resp_ = Upper saturation limit of sigmoid (D), slope = rate of change in the response per unit accommodative demand, Accom_stim_ = the defocus demand used to stimulate a response (D), Accom stim_mid point_ = the midpoint in the defocus stimuli range used in this study.

IQ loss for a given magnitude of induced blur and corresponding accommodative responses were the primary dependent variables in all analyses. The blur stimuli, optical correction modality, and the cohort tested were the independent factors in all analyses. The Shapiro-Wilk test indicated that all dependent variables were normally distributed, excepting few isolated datasets in which normality could not be restored through standard data transformation techniques. Parametric analyses were therefore conducted for all outcomes in this study. *P* ≤ 0.05 was considered statistically significant.

## Results

### Through-Focus Curves

**Figure 3.**
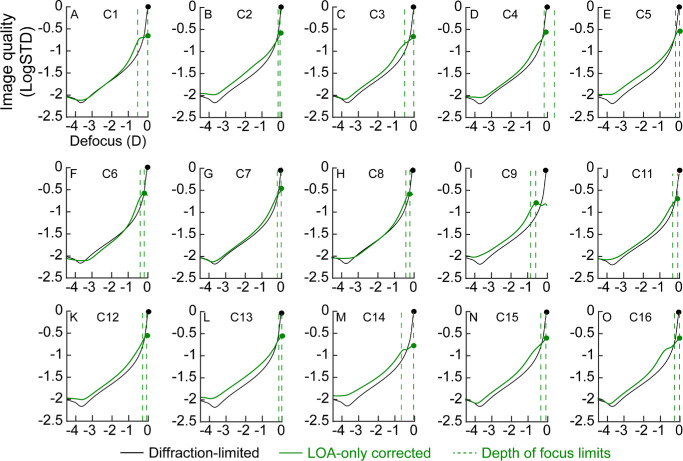
Through-focus curves derived for the logSTD IQ metric for controls in the LOA-only corrected (*green curve*) and LOA + HOA corrected condition (diffraction-limited; *black curve*). Increasing negative values of the ordinate indicates worsening IQ. The *black* and *green filled circles* indicate the peak IQ, and the *vertical green dashed lines* indicate the depth of focus range (i.e. dioptric range between peak IQ and 80% of peak IQ). The participants are labeled in the order of their recruitment into the study.

**Figure 4.**
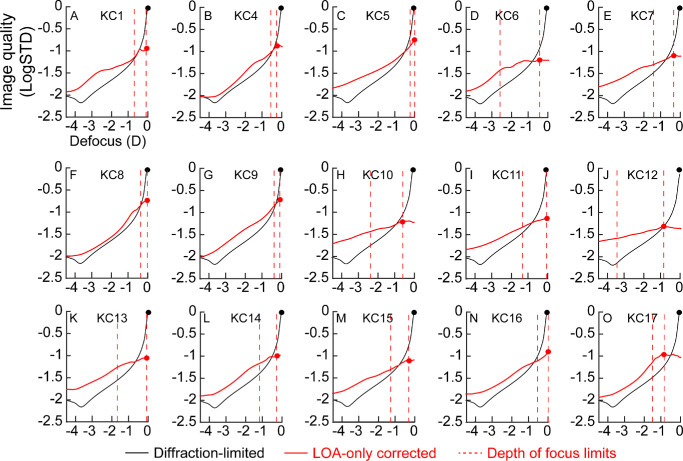
Through-focus curves derived for the logSTD IQ metric for patients with keratoconus in the LOA-only corrected (*red curve*) and LOA + HOA corrected condition (diffraction-limited; *black curve*). Increasing negative values of the ordinate indicates worsening IQ. The *black* and *red filled circles* indicate the peak IQ, and the *vertical red dashed lines* indicate the depth of focus range (i.e. dioptric range between peak IQ and 80% of peak IQ). The participants are labeled in the order of their recruitment into the study.

The through-focus curves of controls in the LOA-only corrected condition showed peak IQ (mean ± 1 SD = −0.61 ± 0.09 logSTD units) occurring at a best-focus of −0.08 ± 0.15 D and with a depth of focus of −0.37 ± 0.22 D (Fig. 3; see Fig. 8 for average ±1-SEM data). The through-focus curves of cases showed larger inter-subject variability than controls, but they were in general flatter (notably, KC6, KC7, KC10 to KC15, and KC17), with poorer peak IQ (−0.99 ± 0.18 logSTD units, *P* < 0.001), more myopic best foci (−0.28 ± 0.34 D, *P* = 0.05) and wider depth of foci (−1.37 ± 0.91 D, *P* < 0.001; see Fig. 4; see Fig. 8 for average ±1-SEM data). The ANOVA showed significant main effects of blur magnitude and cohort on the IQ loss (Table 2, Section 1a). The IQ loss for each blur magnitude was significantly different from the adjacent values (Table 2, Section 1b). The interaction between factors was also significant, indicating non-uniform IQ loss with increasing blur across cohorts (see Table 2, Section 1a).

**Table 2.** Results of the Statistical Analyses Conducted in This Study

Section 1: Two-Factor Mixed Effect ANOVA				
Impact of Induced Defocus on IQ Loss in Controls and Keratoconic Cases				
**1a. Multivariate Test**				
		**F**	***P*** **Value**	**Partial ƞ^2^**

Blur		101.58	**<0.001**	0.96
Cohort		60.13	**<0.001**	0.68
Blur × Cohort		18.05	**<0.001**	0.79
**1b. Univariate Test**				

			**Mean Diff ± 1 SEM**	***P*** **Value**

Blur	0.5 D	1.0 D	−0.33 ± 0.03	**<0.001**
		1.5 D	−0.59 ± 0.04	**<0.001**
		2.0 D	−0.81 ± 0.05	**<0.001**
		3.0 D	−1.19 ± 0.06	**<0.001**
		4.0 D	−1.45 ± 0.07	**<0.001**
	1.0 D	1.5 D	−0.27 ± 0.02	**<0.001**
		2.0 D	−0.48 ± 0.02	**<0.001**
		3.0 D	−0.87 ± 0.04	**<0.001**
		4.0 D	−1.12 ± 0.05	**<0.001**
	1.5 D	2.0 D	−0.21 ± 0.01	**<0.001**
		3.0 D	−0.60 ± 0.03	**<0.001**
		4.0 D	−0.85 ± 0.04	**<0.001**
	2.0 D	3.0 D	−0.38 ± 0.02	**<0.001**
		4.0 D	−0.64 ± 0.03	**<0.001**
	3.0 D	4.0D	−0.26 ± 0.02	**<0.001**
Cohort	Controls		1.48 ± 0.10	**<0.001**
	Cases		0.47 ± 0.09	
**Section 2: Three-Factor Mixed Effect ANOVA**				
**Impact of Induced Defocus on the Accommodative Response in Controls and Keratoconus Cases Under LOA-Only Corrected and LOA + HOA Corrected Conditions**				
**2a. Multivariate Test**				
** **		**F**	***P*** **Value**	**Partial ƞ^2^**

Blur		35.68	**<0.001**	0.88
Correction		0.32	0.578	0.01
Cohort		35.74	**<0.001**	0.56
Blur × Cohort		7.92	**<0.001**	0.62
Correction × Cohort		0.13	0.726	0.01
Blur × Correction		0.06	0.998	0.01
Blur × Correction × Cohort		0.34	0.884	0.07
**2b. Univariate Test**				
			**Mean Diff ± SEM**	***P*** **Value**

Blur	0.5 D	1.0 D	−0.28 ± 0.05	**<0.001**
		1.5 D	−0.48 ± 0.07	**<0.001**
		2.0 D	−0.78 ± 0.07	**<0.001**
		3.0 D	−1.35 ± 0.10	**<0.001**
		4.0 D	−1.59 ± 0.16	**<0.001**
	1.0 D	1.5 D	−0.19 ± 0.06	0.080
		2.0 D	−0.49 ± 0.08	**<0.001**
		3.0 D	−1.07 ± 0.08	**<0.001**
		4.0 D	−1.30 ± 0.16	**<0.001**
	1.5 D	2.0 D	−0.30 ± 0.07	**0.001**
		3.0 D	−0.87 ± 0.09	**<0.001**
		4.0 D	−1.11 ± 0.14	**<0.001**
	2.0 D	3.0 D	−0.58 ± 0.08	**<0.001**
		4.0 D	−0.81 ± 0.12	**<0.001**
	3.0 D	4.0 D	−0.24 ± 0.11	0.71
Cohort	Controls		1.42 ± 0.10	**<0.001**
	Cases		0.55 ± 0.10	

**Table 2.** Continued

**Section 3: Two-Factor Repeated Measure ANOVA**				
**Effect of Induced Keratoconic Blur on Accommodative Response in Controls Compared to Responses With Baseline LOA-Only Corrected Condition**				
**3a. Multivariate Test**				
		**F**	***P*** **Value**	**Partial ƞ^2^**

Blur		40.05	<0.001	0.98
Correction		22.04	0.001	0.71
Blur × Correction		9.51	0.014	0.91
**3b. Univariate Test**				
			**Mean Diff ± SEM**	***P*** **Value**

Blur	0.5 D	1.0 D	−0.18 ± 0.07	0.36
		1.5 D	−0.54 ± 0.08	**0.002**
		2.0 D	−0.74 ± 0.08	**<0.001**
		3.0 D	−1.53 ± 0.11	**<0.001**
		4.0 D	−1.94 ± 0.17	**<0.001**
	1.0 D	1.5 D	−0.35 ± 0.11	0.13
		2.0 D	−0.56 ± 0.13	**0.02**
		3.0 D	−1.35 ± 0.13	**<0.001**
		4.0 D	−1.75 ± 0.19	**<0.001**
	1.5 D	2.0 D	−0.20 ± 0.09	0.88
		3.0 D	−0.99 ± 0.12	**<0.001**
		4.0 D	−1.40 ± 0.18	**<0.001**
	2.0 D	3.0 D	−0.79 ± 0.09	**<0.001**
		4.0 D	−1.19 ± 0.12	**<0.001**
	3.0 D	4.0 D	−0.41 ± 0.17	0.65
Cohort	Baseline		1.70 ± 0.08	**0.001**
	Induced		0.79 ± 0.26	

Relationships with *P* < 0.05 (corrected for multiple comparisons) appear in bold.

The effect size is indicated by the partial ƞ^2^ value.

### Accommodative Stimulus-Response Function With LOA-Only and LOA + HOA Correction

**Figure 5.**
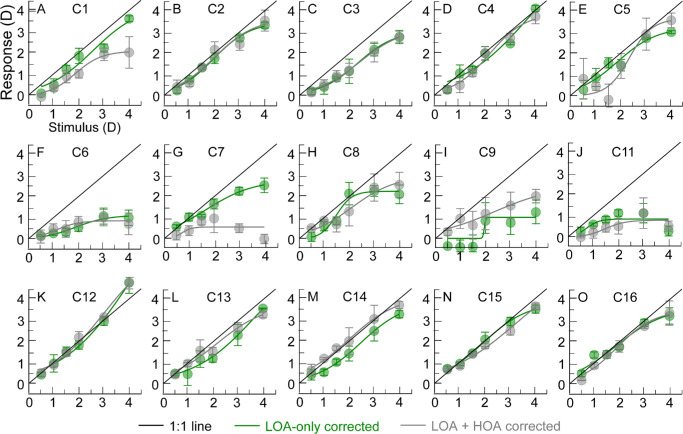
The accommodative stimulus-response function for controls in the LOA-only corrected (*green data*) and LOA + HOA corrected condition (diffraction-limited; *gray data*). Each data point is an average of the three measurements, and the error bar represents the ±1 standard deviation. The curves represent the best-fit sigmoid function to the data (see Equation 2 for details). The black 1:1 line in each panel indicates the accommodative response equaling the stimulus. The order of participant labeling is identical to Figure 3.

**Figure 6.**
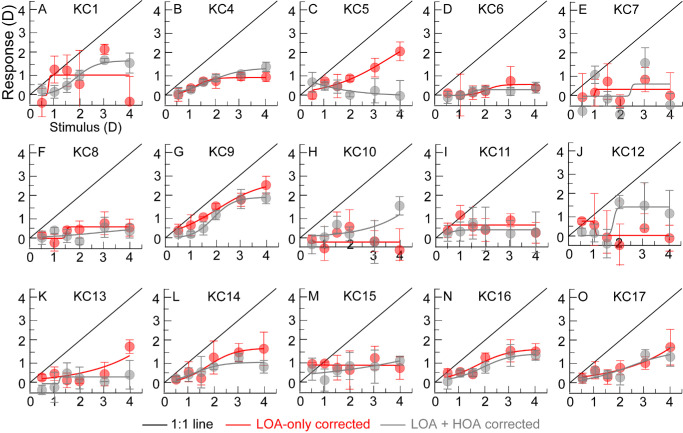
The accommodative stimulus-response function for patients with keratoconus in the LOA-only corrected (*red data*) and LOA + HOA corrected condition (diffraction-limited; *gray data*). Each data point is an average of the three measurements, and the error bar represents the ±1 standard deviation. The curves represent the best-fit sigmoid function to the data (see Equation 2 for details). The black 1:1 line in each panel indicates the accommodative response equaling the stimulus. The order of participant labeling is identical to Figure 3.

**Figure 7.**
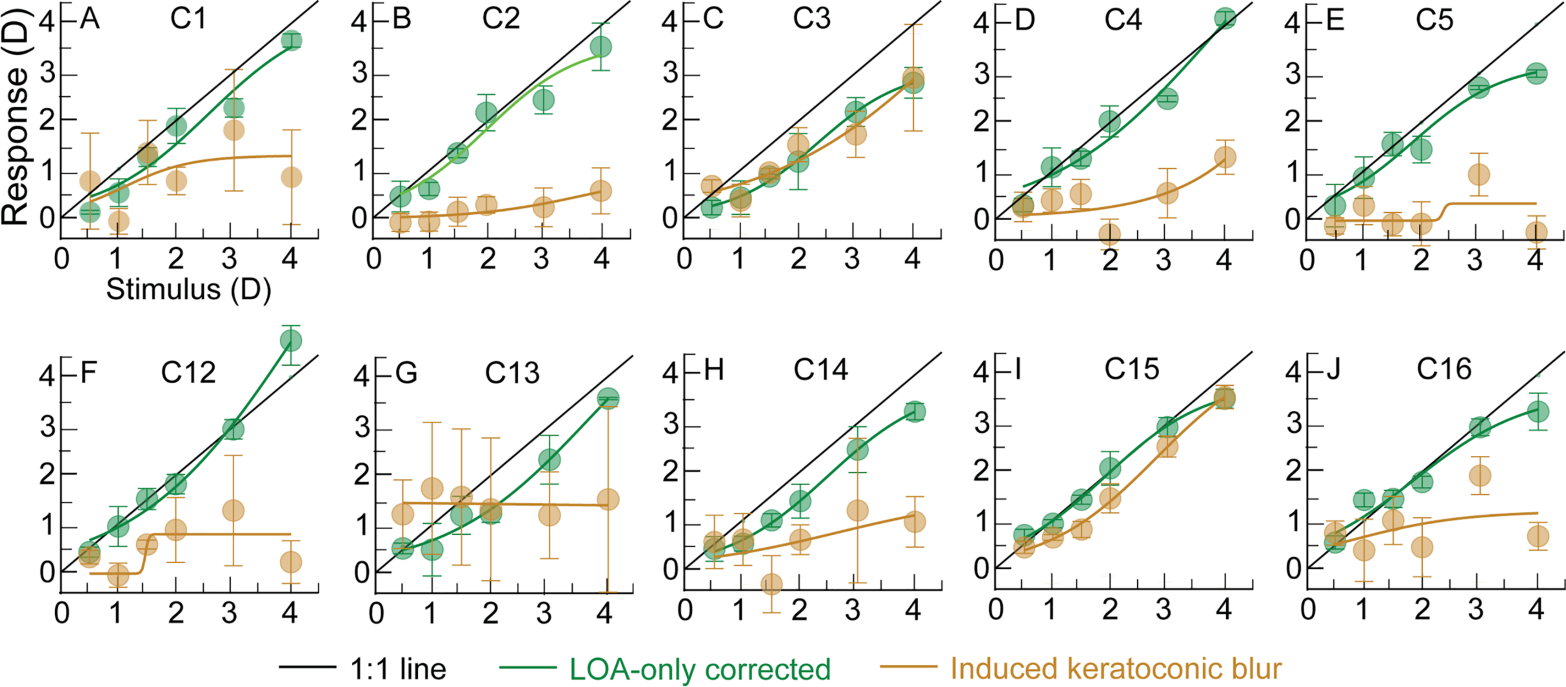
The accommodative stimulus-response functions of the 10 control participants on whom the keratoconic HOAs were induced to manipulate the eye's optical quality. The *green* and *gold symbols* indicate data from the baseline LOA-only corrected condition and the induced keratoconic blur condition, respectively. All other details are the same as Figure 5.

**Figure 8.**
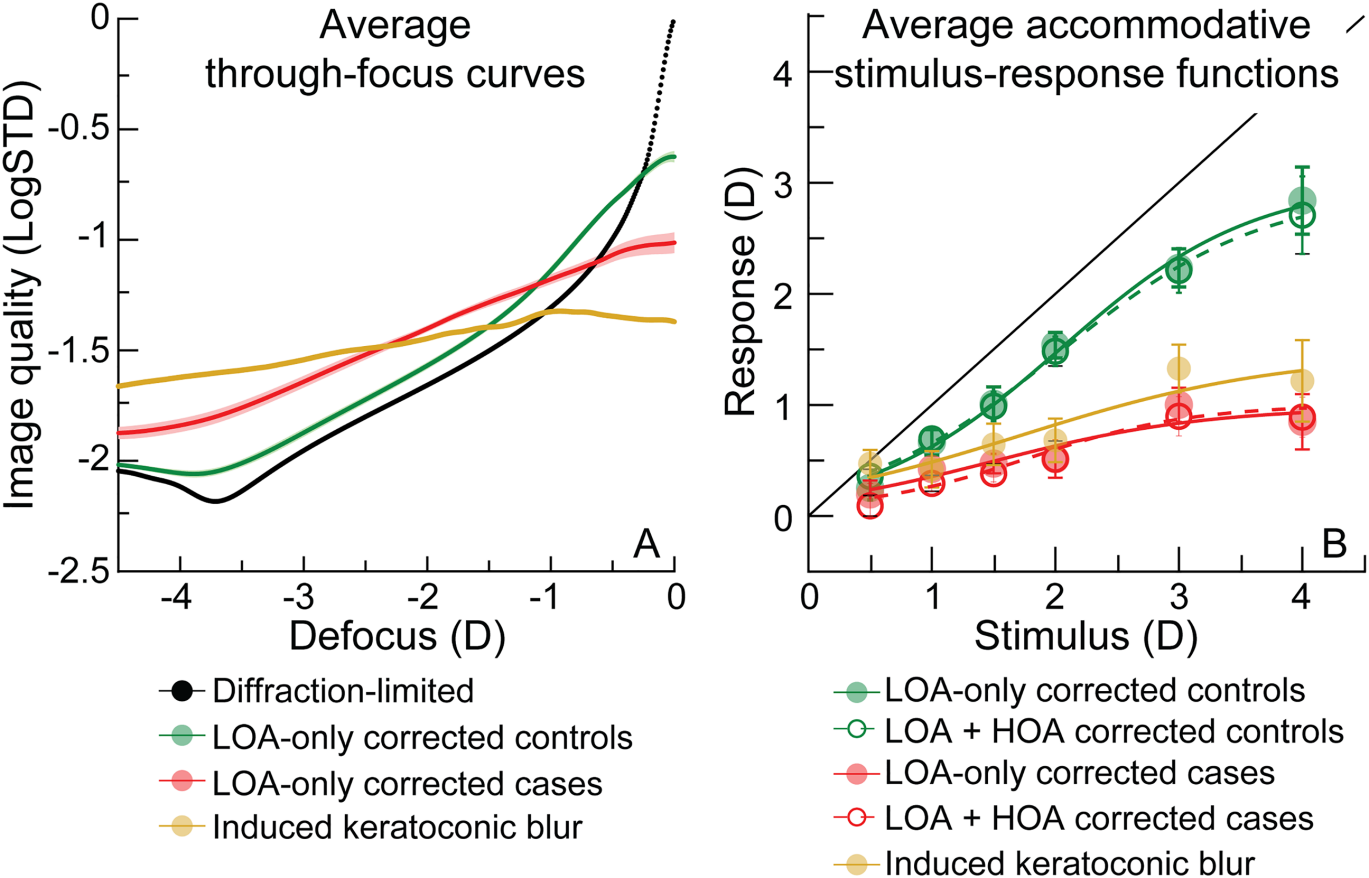
(**A**) The average (±1 SEM) through-focus curve across controls (*green curve*) and cases (*red curve*) under the LOA-only corrected condition in this study. Also shown is the diffraction-limited through focus curve (*black curve*) and the through-focus curve of the keratoconic case whose HOA profile was used to induced blur in controls (*gold curve*). (**B**) The average (±1 SEM) accommodative stimulus-response function of cases and controls under the LOA-only corrected condition (*closed symbols with solid traces*) and LOA + HOA corrected condition (*open symbols with dashed traces*). The average data of controls with induced HOAs from one keratoconic case is also shown here for comparison purposes.

Controls showed steep sigmoid-shaped stimulus-response functions in most participants in the LOA-only corrected condition (Fig. 5; see Fig. 8 for average ±1-SEM data). Three controls (C6, C9, and C11) showed relatively attenuated stimulus-response functions while one control (C12) showed accommodative lead for the largest blur stimulus (see Fig. 5). The stimulus-response functions in the LOA + HOA corrected condition were similar to those in the LOA-only corrected condition in most controls (see Fig. 5). In participants C1 and C7, the data in the LOA + HOA corrected condition was attenuated more than the LOA-only corrected condition (see Fig. 5). This trend reversed in participant C9 (see Fig. 5). The stimulus-response functions in the LOA-only corrected condition was attenuated in most cases (Fig. 6; see Fig. 8 for average ±1-SEM data). Among them, participants KC5, KC9, KC14, KC16, and KC17 were relatively steep functions with the response range reaching approximately 2.25 D across the entire stimulus range (see Fig. 6). Like the controls, the data in the LOA + HOA corrected condition were similar to the LOA-only corrected condition, with participants KC1, KC10, and KC12 showing steeper functions in the former than latter condition (Fig. 7). This trend reversed for participants KC5, KC13, and KC14 (see Fig. 7). The main effects of blur magnitude and cohort on the accommodative response was statistically significant (see Table 2, Section 2a). The interaction between factors was also significant, reflecting differences in the slopes of the stimulus-response functions between cohorts (see Figs. 5, 6, 8). Post hoc testing confirmed that the accommodative response for each blur stimulus was significantly different from the adjacent value, barring a few pairwise comparisons noted in Table 2, Section 2b. The main effect of correction modality and other interactions were not significant.

### Accommodative Responses in Controls With Induced Keratoconic HOAs

Eight participants showed attenuated accommodative responses with induced keratoconic HOAs, with the overall range of accommodation restricted to within 1.5 D (see Fig. 7; see Fig. 8 for average ±1-SEM data). The two remaining participants (C3 and C15) showed no change in accommodative performance (see Fig. 7). The main effects of blur magnitude and correction modality on the accommodative response was statistically significant (Table 2, Section 3a). The interaction between the factors was also significant, reflecting the differences in the slopes of the accommodative stimulus-response function between the two correction modalities. Post hoc testing confirmed that the accommodative response for each blur value was significantly different from the adjacent values, barring a few pairwise comparisons (Table 2, Section 3b).

### Correlation Among IQ Loss, Accommodative Response, and Disease Severity

**Figure 9.**
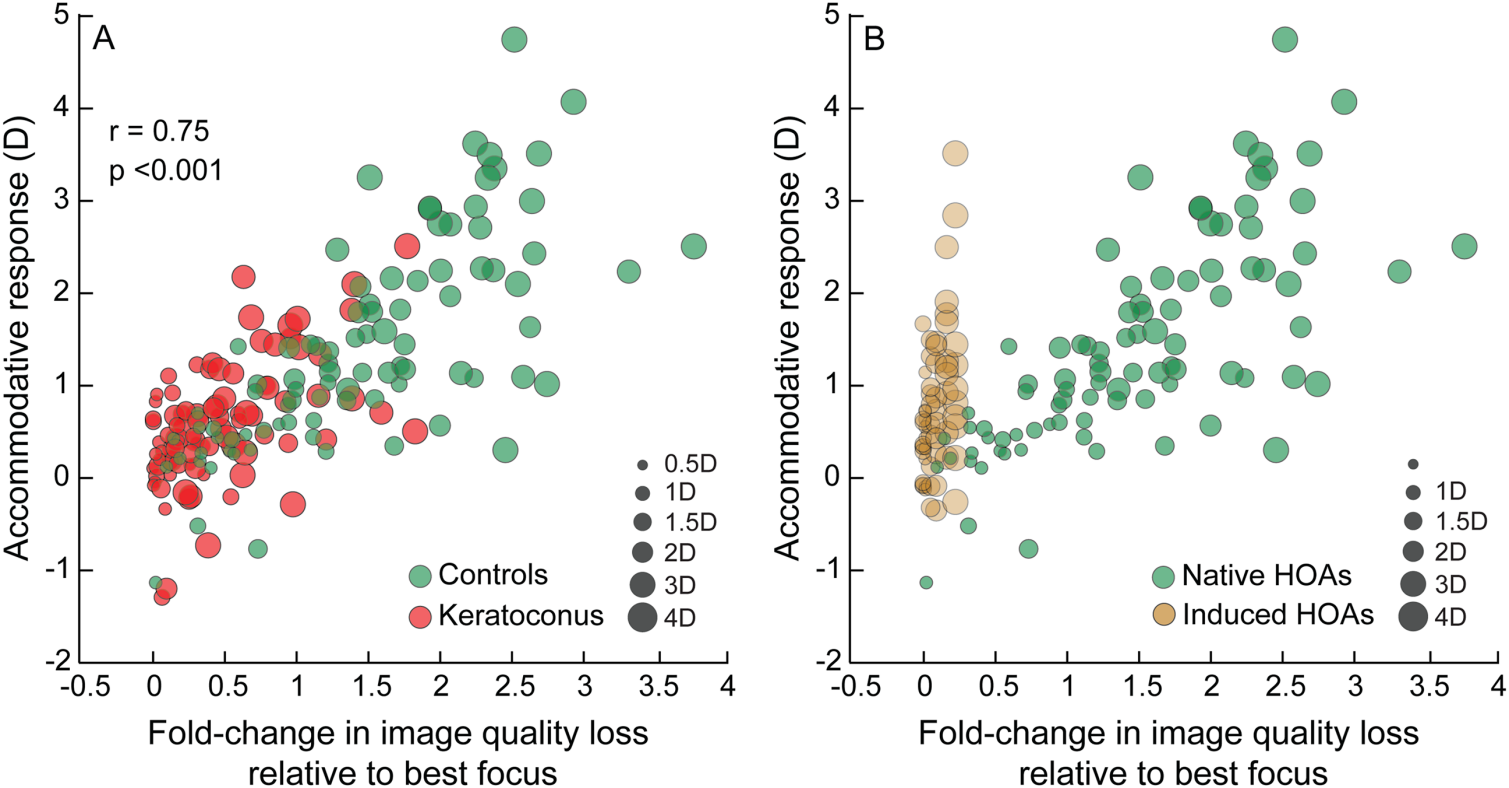
(**A**) Bubble plots showing the relationship between IQ loss with induced blur and the corresponding accommodative responses in controls and cases. (**B**) Similar plot showing data of controls with native HOAs (i.e. LOA-only corrected condition) and with induced keratoconic HOAs. The bubble size is scaled to the magnitude of induced blur, as shown in the figure.

Figure 9A shows bubble plots of the accommodative response plotted against the corresponding IQ loss derived from the through-focus curves. The data distribution of controls showed larger magnitudes of blur to generate larger losses in IQ. The overall range of IQ loss was restricted in cases, relative to controls. A positive correlation was observed between the variables (*r* = 0.75, *P* <0.001), indicating a strong association between the IQ loss and accommodative responses. Controls C6, C9, and C11 were exceptions to this general relationship, for their accommodative responses were attenuated even though the corresponding through-focus curves showed a robust loss in IQ with induced blur (compare panels F, I, and J in Figs. 3 and 5). That there may be a cause-effect relationship between the IQ loss and accommodative responses is reinforced in Figure 9B, wherein the induction of keratoconic HOAs in controls resulted in a significant attenuation of IQ loss and accommodative responses.

The D-index was significantly negatively correlated with the change in IQ loss between 0.5 D and 4 D (*r* = −0.85, *P* < 0.001) as well as with the change in accommodative response between the two blur stimuli (*r* = −0.66, *P* < 0.001). The correlation coefficient between the D-index and accommodative response was relatively weaker because of the same three control participants noted above (C6, C9, and C11). The correlation improved to −0.80 (*P* <0.001) after excluding these participants.

## Discussion

### Summary of Present Results and Practical Implications

The study had three key findings. First, monocular blur-driven accommodation is attenuated in keratoconus, relative to age-similar controls. This loss is more prominent for large accommodative demands and for participants with greater severity of keratoconus. Second, strengthening the blur signal by correcting HOAs does not result in any significant change in the accommodative response of both cohorts. Third, weakening the blur signal in controls by inducing keratoconic HOAs significantly worsens their accommodative responses. Overall, to the best of the authors’ knowledge, these results are the first direct demonstration of deficiency in monocular, blur-driven accommodation being governed by the eye's optical quality in keratoconus. These results, however, resonate well with Yildiz et al.^13^ who demonstrated large lags of accommodation in keratoconic individuals wearing two different types of rigid contact lenses and with Ohmi et al.^11^ who found the maximum amplitude of accommodation to be lower in keratoconus than in controls. The present results are also in alignment with Gambra et al.^6^ who showed reduced accommodative gain with induced positive spherical aberration and coma in individuals with physiologically normal optics.

These results indicate that keratoconic individuals may not effectively use blur-driven accommodation to meet their near vision demands.^33^ In fact, qualitative difficulties in near-vision activities (e.g. reading a menu card or sheet music),^34^ degraded depth-related visuomotor task performance at near viewing,^35^ and visual discomfort from deficient accommodative/binocular vision functions^36^ have all been documented in keratoconus. All these may collectively worsen the quality of life in this disease condition.^15^^,^^37^ Thus, a comprehensive evaluation of the accommodative and binocular vision status of the patient with keratoconus is critical for identifying such issues and mitigating them with appropriate management strategies.

### Relationship Between Image Quality and Blur-Driven Accommodation

Monocular blur-driven accommodation may be governed by the quality of blur signal available for visual processing – the steeper the through-focus curve, the better is the reliability of blur signal for driving accommodation and steeper the accommodative stimulus-response function (see Fig. 1). In other words, even while the accommodative demands may be equal between controls and cases in this study, these translate in lower magnitudes of blur signal in cases owing to their already blurred IQ (see Fig. 1B). In eyes with physiologically normal optics or with mild keratoconus (e.g. participants KC9 and KC14 with the lowest D-index and, therefore, the least severe keratoconus in this study; see Table 1), a strong accommodative signal may be generated by the loss of energy at the mid and high spatial frequency range with induced blur,^38^ which may then be restored using the blur-driven negative feedback mechanism (Figs. 4 and 6).^39^^,^^40^ The contrast energy of higher spatial frequencies diminishes below the neural detection thresholds with increasingly aberrated optics, leaving only a weaker accommodative signal to be generated from the available low spatial frequencies.^38^ This was the case for participants KC1, KC6 to 8, KC10 to 12, and KC15, who had relatively more severe forms of keratoconus in this study (see Table 1) and whose through-focus curves were significantly flatter than those of controls (see Fig. 4). The availability of only low spatial frequency information for visual processing in these eyes effectively renders blur open-loop,^38^^,^^41^ resulting in relatively flat accommodative stimulus-response functions (see Fig. 6). This effect is expected to be similar or more pronounced for eyes with increasing severities of keratoconus, even though they were not explicitly investigated here.

Image quality loss with blur is characterized by both contrast losses and phase shifts.^42^^,^^43^ Although the aforesaid discussion revolves around the contrast energy available for driving accommodation, the impact of phase shifts arising in keratoconic eyes with highly aberrated optics may not be ignored.^42^^,^^43^ The exaggerated spherical aberration, coma, and trefoil in keratoconus are known to induce doubling/ghosting of the visual image, as apparent in the Maltese cross image shown in Figure 1B of this study. Presently, it is not clear which signal in the blurred image – contrast loss or phase shifts or both – the visual system uses to generate an accommodative response. A detailed analysis of the impact of each aberration component on the contrast and phase spectrum is necessary to gain insights into this issue.

### Sufficiency of Optical Blur as a Cue for Accommodation

Three observations suggest that the blur signal strength may not be the only factor influencing accommodative performance in this study. First, three controls (C6, C9, and C11) and three cases (KC4, KC8, and KC 14) generated shallower accommodative stimulus-response functions despite having comparable image qualities as peers with steep stimulus-response functions (see Figs. 4–7). Second, the accommodative responses of two controls (C1 and C7; see Fig. 5) and three cases (KC5, KC13, and KC14; see Fig. 6) worsened with the correction of HOAs, relative to when they were left intact. Third, one control showed an accommodative lead for large blur stimuli (C12; see Fig. 5) and the accommodative responses of two controls did not change with induced keratoconic HOAs (C3 and C15; see Fig. 5). Given that the blur stimuli were similar in all these individuals, it is possible that they were accustomed to using cues other than blur to drive accommodation (e.g. retinal disparity,^44^ target proximity and looming,^45^ and volition^46^). Indeed, there is evidence for these sensory cues to be weighted differently across individuals and for retinal disparity to be the strongest among the individual cues to drive accommodation in those with physiologically normal optics.^1^^,^^47^^–^^49^ The cue impoverishment that arose from the absence/paucity of these cues in the present study perhaps led to worsened accommodative performance in the aforesaid individuals/conditions.

There was no evidence of an improvement in the accommodative performance with the correction of LOAs and HOAs in this study. The results on controls are similar to the previous observation of a limited improved accommodative gain with full correction of wavefront aberrations in participants with physiologically normal optics.^50^^,^^51^ The magnitude of HOAs is usually small in controls and, perhaps, their removal did not result in any major value addition for accommodation.^30^^,^^52^ In contrast, there may be larger room for improvement in accommodation of keratoconic cases with HOA correction (see Fig. 1C). That such an advantage was not observed in the present study may either reflect neural insensitivity to blur arising from chronically degraded optics in keratoconus^10^ or to the novelty of the HOA-corrected stimulus that the keratoconic visual system is yet to get calibrated to.^53^ The former inference is in line with the observations of Sabesan and Yoon (2009) who found limited improvement in the visual acuity of keratoconic eyes corrected for their HOAs using an adaptive optics set-up, vis-à-vis, controls. This suggests that long term visual experience with poor retinal image quality may impact not only high-spatial frequency mediated resolution tasks but also oculomotor responses mediated by intermediate spatial frequencies.^54^ Pupil miosis at near distances could also compensate for deficient accommodation in keratoconus by reducing the impact of aberrated optics.^55^ Previous studies have reported smaller mesopic pupil diameters^56^ and a shift in the pupil center^57^ in keratoconus, suggesting an adaptation to optimize IQ in the presence of exaggerated wavefront aberrations. Recent evidence also points to altered thickness and vasculature of the retina and choroid in keratoconus, relative to age-similar controls.^58^^–^^60^ These structural changes may also contribute to the degraded accommodative responses in keratoconus, should they alter the contrast processing capabilities of the retina. Future studies are required to address these speculations systematically.

### Study Limitations

The present study had three limitations. First, the dynamic trajectory of the accommodative response could not be tracked in this study. Such an assessment in the future would offer deeper insights into how the keratoconic visual system may plan and execute these motor responses amidst degraded image quality. Second, the far point of participants was determined from objective sphero-cylindrical values using the standard convention of maximum plus power for maximum visual acuity, without an explicit attempt to relax accommodation for distance viewing (e.g. using duochrome test or other “fogging” techniques). This imprecision may reflect in the small negative values of accommodation recorded at the 0 D blur stimulus in some participants (see Figs. 5, 6). Third, image quality was determined for 555 nm of light, but the visual targets remained polychromatic to ensure the presence of longitudinal chromatic aberration as an odd-error cue for accommodation. For this reason, direct comparison of the computational IQ results with empirical accommodation is challenging. Construction of polychromatic image quality may address this issue in the future.^61^ The latter two limitations, combined with the fact that small changes in spherical aberration were ignored in the determination of accommodation (Appendix I), render the estimation of absolute accommodative states ambiguous.^62^^–^^64^ Caution should therefore be exercised if inferences about accommodative lags/leads are made from the data shown in this study. However, these limitations are expected to be uniform across cohorts, leaving the overall study conclusions unaffected.

## Conclusions

Monocular, blur-driven accommodation is attenuated in keratoconus, relative to non-keratoconic controls. This result is consistent with a weakened defocus-blur signal available to drive accommodation. The absence of improvement in accommodative performance despite optical quality enhancement with correction of LOAs and HOAs may indicate a limited role of pure defocus as an accommodative cue and/or reduced neural sensitivity to blur following chronic exposure to degraded image quality.

## Figures and Tables

**Figure A1.**
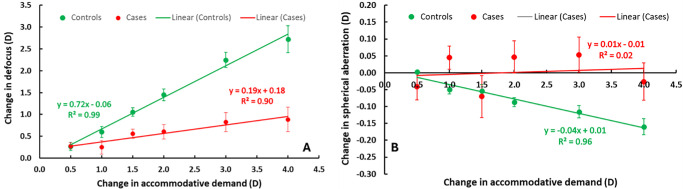
(**A**) Mean (±1 SEM) change in defocus term of the Zernike polynomial series measured over a 4.5-mm pupil diameter plotted as a function of the change in accommodative demand for controls (*green data*) and cases (*red data*) that participated in the study. This data is plotted as the accommodative stimulus response function in Figure 8 of the revised manuscript. The defocus values were converted from microns into equivalent diopters using Equation A1. (**B**) Similar data for spherical aberration (SA). The spherical aberration values were converted from microns into equivalent diopters using Equation A2.

## Appendix I. Appendix I. Changes in defocus and spherical aberration with accommodative demands in controls and cases

Accommodative responses to changes in target vergence are associated with changes in both defocus and spherical aberration.^29^^–^^32^ The magnitude of change in spherical aberration is also dependent on the level of accommodative demand/response.^29^^–^^32^ To determine if a similar trend was observed in this study, the Zernike aberration terms corresponding to defocus and spherical aberration estimated from the Shack-Hartmann sensor were analyzed for the different accommodative demands in controls and cases (Fig. A1). These terms were converted from microns into diopters using Equations A1 and A2, for defocus and spherical aberration, respectively.^25^ The mean (±1 SEM) defocus (*green* datapoints in panel **A**; already reported in the main text as accommodative responses) and spherical aberration (*green* datapoints in panel **B**) changed linearly with increasing accommodative demands in controls. The defocus term increased positively with accommodative demand, indicating an overall increase in the eye's optical power, as would be expected from an accommodative response. The spherical aberration term became more negative with increasing accommodative demands, as reported in the literature.^62^^,^^64^ The linear regression slopes for spherical aberration was approximately 1/14th the steepness of the defocus slope, confirming that defocus changes remained the dominant change with increasing accommodative demands. The data of keratoconic cases showed an overall shallower slope of changes in defocus, reflecting the weaker accommodative response in this cohort, relative to controls (*red* datapoints in panel **A**). Spherical aberration did not show any meaningful change in this cohort (*red* datapoints in panel **B**).

(A1)
$$\begin{array}{ccc} & & \mathrm{Defocus}\;in\;\mathrm{diopters}=\left(4*pi\;*\;\sqrt{3}\right)\\ & & \;\;*\;\left(\mathrm{defocus}\;in\;\mathrm{microns}/\mathrm{pupil}\;\mathrm{area}\right) \end{array}$$

(A2)
$$\begin{array}{ccc} & & Spherical\;\mathrm{aberration}\;in\;\mathrm{diopters}\;\\ & & \;=(24\;*\;\sqrt{5})/(pupil\;{radius}^{\wedge 4})\\ & & \;*(\mathrm{Spherical}\;\mathrm{aberration}\;in\;\mathrm{microns}) \end{array}$$
